# Nutrient Intakes Linked to Better Health Outcomes Are Associated with Higher Diet Costs in the US

**DOI:** 10.1371/journal.pone.0037533

**Published:** 2012-05-25

**Authors:** Anju Aggarwal, Pablo Monsivais, Adam Drewnowski

**Affiliations:** 1 Center for Public Health Nutrition, School of Public Health, University of Washington, Seattle, Washington, United States of America; 2 Center for Human Nutrition, Department of International Health, Johns Hopkins Bloomberg School of Public Health, Baltimore, Maryland, United States of America; 3 United Kingdom Clinical Research Collaboration Centre for Diet and Activity Research, Cambridge Institute of Public Health, Cambridge, United Kingdom; Indiana University, United States of America

## Abstract

**Purpose:**

Degrees of nutrient intake and food groups have been linked to differential chronic disease risk. However, intakes of specific nutrients may also be associated with differential diet costs and unobserved differences in socioeconomic status (SES). The present study examined degrees of nutrient intake, for every key nutrient in the diet, in relation to diet cost and SES.

**Methods:**

Socio-demographic data for a stratified random sample of adult respondents in the Seattle Obesity Study were obtained through telephone survey. Dietary intakes were assessed using food frequency questionnaire (FFQ) (n = 1,266). Following standard procedures, nutrient intakes were energy-adjusted using the residual method and converted into quintiles. Diet cost for each respondent was estimated using Seattle supermarket retail prices for 384 FFQ component foods.

**Results:**

Higher intakes of dietary fiber, vitamins A, C, D, E, and B12, beta carotene, folate, iron, calcium, potassium, and magnesium were associated with higher diet costs. The cost gradient was most pronounced for vitamin C, beta carotene, potassium, and magnesium. Higher intakes of saturated fats, trans fats and added sugars were associated with lower diet costs. Lower cost lower quality diets were more likely to be consumed by lower SES.

**Conclusion:**

Nutrients commonly associated with a lower risk of chronic disease were associated with higher diet costs. By contrast, nutrients associated with higher disease risk were associated with lower diet costs. The cost variable may help somewhat explain why lower income groups fail to comply with dietary guidelines and have highest rates of diet related chronic disease.

## Introduction

Observational studies on diets and health have linked the consumption of individual nutrients with chronic disease risk [1]–[13]. Lower intakes of dietary fiber [14]–[19], folate [8], [20], carotenoids [21], vitamins A, C, and E [7], calcium [20], [22], [23], and potassium [19], [24], [25] have all been linked with a higher risk of chronic disease in a dose-dependent manner [26]. By contrast, higher intakes of sugars, saturated fats and trans fats have been linked with a higher risk of heart disease [27]–[30], obesity, and diabetes [31]–[33]. In many such studies, nutrient intakes were adjusted for energy using the method of residuals and the participants were divided into quintiles [6]–[8], [10], [13], [17], [19], [21], [30]. To account for the possibility that people with higher nutrient intakes differed in profound yet unobserved ways from those with lower nutrient intakes, many studies adjusted for socioeconomic status (SES) using proxy indicators such as smoking and physical activity [1], [2], [4], [6], [7], [17], [24], [25]. Promoting the consumption of beneficial nutrients while limiting the intake of added sugars and fats is a standard dietary guidance in US [26], [34].

However, such dietary guidelines may not be feasible until factors underlying differential nutrient intakes are taken into account. Once such potential factor may be diet cost. Recent studies, mostly from outside US, have found links between nutrient intakes and estimated individual-level diet cost [35]–[39]. Lower energy-adjusted diet cost was associated with lower intakes of protein, fiber, and some key vitamins and minerals, raising the issues of nutrient adequacy of diets of lower-income groups. Within the US, there are limited data on the relation between diet quality and diet cost, particularly at the level of every nutrient in the diet [40], [41]. Further, there has been a controversy whether lower cost diets are truly selected by lower income groups.

The purpose of the present study was to examine if degree of nutrient intakes commonly associated with lower disease risk and improved health outcomes would be associated with higher diet costs. Conversely, whether degree of nutrient intakes commonly associated with adverse health outcomes would be associated with lower diet cost. The cost gradient might help explain why lower income groups have least nutrient adequate diets and are at higher risk for chronic disease including obesity and diabetes.

The present study followed standard procedures of estimating and defining each nutrient intake [42]. Nutrient intakes were adjusted for energy using the method of residuals and the participants were divided into quintiles. The primary hypotheses were that the recommended nutrients, notably dietary fiber, vitamins and minerals, would be associated with higher diet costs whereas nutrients to limit, notably sugars and fat, would be associated with lower diet costs. The secondary hypothesis was that lower-cost lower-quality diets would be more likely to be consumed by lower SES respondents.

## Methods

### Ethics Statement

All the study protocols and study instruments were approved by Institutional Review Board (IRB) at University of Washington.

### Participant Sample

Methods and procedures for the Seattle Obesity Study (S.O.S.) have been published [43], [44]. The S.O.S. used a stratified area-based sampling to ensure adequate representation by income range and race/ethnicity. The sampling procedures and survey administration were modeled on the Behavioral Risk Factors Surveillance System (BRFSS) telephone surveys conducted by state and local health departments. Randomly generated telephone numbers were matched with residential addresses using commercial databases. All the potential respondents were sent a pre-notification letter followed by the telephone call. Once the contact was made, an adult member of the household was randomly selected as the respondent using CATI (computer assisted telephone interview) program. All the study procedures were explained to the selected respondent and a verbal consent to participate in the study was obtained. A 20 min telephone survey was then administered to 2,001 study respondents by trained and computer assisted interviewers.

#### Dietary intake assessment

At the completion of the telephone survey, each of the 2,001 respondents was asked for their verbal consent to complete a written dietary questionnaire. Both the scripts used to obtain verbal consent for completing the telephone survey and FFQ were provided to IRB at the University of Washington and were approved. 95% of the survey participants (n = 1,903) who agreed were mailed a food frequency questionnaire (FFQ) developed by the Fred Hutchinson Cancer research Center (FHCRC) [45]–[48]. Participants recorded the frequency of consumption of foods and beverages listed in the FFQ along with the portion size. Completion rate was 69% (n = 1,318). Of these, 52 questionnaires were excluded based on extreme calorie intakes (<500 or >5000 kcal/day) and other missing data leaving a final sample of 1,266 (804 women and 462 men). FFQ respondents were compared to those who did not respond to FFQs. FFQ respondents were more likely to married (53% vs. 45%), college-educated (57% vs. 51%) and retired (28% vs.14%). No significant differences were seen by other SES characteristics, diet variables or health outcomes.

Analyses of dietary data obtained from FFQ yielded dietary energy (kcal), the weight of foods, beverages, and drinking water (g), and the estimated daily intakes of over 45 macro- and micronutrients. Data from all foods and caloric beverages was used for present analyses. Details on computation of these variables from FFQ have been published [40].

### Measures of dietary intake

Nutrients of interest were vitamins A, C, D, E, B12, beta carotene, folate iron, calcium, potassium, magnesium, and fiber. Following past studies, the nutrient residual model was used to adjust for energy [49] and study participants were then split into 5 equal groups or quintiles [3], [7], [8], [13], [19], [21]. Intakes of saturated fats, trans fat and added sugar were expressed as percent calories and participants were divided into quintiles.

The monetary value of the reported diets ($/day) was then calculated for each respondent. Lowest retail price at which a given food item was available in the area was attached to each of the 384 component foods in the FFQ database. Most of the respondents (88%) reported using supermarkets as their primary store for food shopping. Hence, prices were collected from three most commonly reported supermarkets, which also represented over 60% of the retail grocery market in the area. The detailed methodology has already been published [40], [50]. All food prices were adjusted for yield, that is preparation and waste, and were expressed in dollars per 100 g of edible portion [51]. The USDA had used analogous procedures to create the Center for Nutrition Policy and Promotion food prices database [52]

### Demographic and Socioeconomic Measures

Self-reported data on age, gender, race/ethnicity and household size were obtained during the phone survey. Education was measured in 6 categories ranging from “never attended” to “college graduates”. Income was measured from “less than $10,000” to “greater than $100,000” per year. For analytical purpose, higher education was defined as college graduates or higher. Higher income was defined as those with annual household income ≥50,000. Past studies noted that income and education reflected different aspects of SES and were not a proxy for each other [44], [53]–[55]. Hence, an index of both income and education was created as an indicator of SES. Higher SES was defined as those with annual household income ≥50,000 and who were at least college graduates.

### Statistical Analyses

Multivariable regressions with robust standard errors were used to examine associations of degree of nutrient intakes with diet cost. For vitamins and minerals, dietary intakes were energy-adjusted using the residual method and then converted into quintiles. For fats and added sugar, percent of total calories obtained from each were computed and then converted into quintiles. Covariates included age, gender, race/ethnicity and total calorie intake. Mean diet cost was expressed at mean age (56 years) and mean calorie intake (1800 Kcal/d) for the sample. Trend tests were conducted. These analyses were also repeated after stratifying by gender. All the diet cost values obtained were standardized at 100% of the bottom quintile for each nutrient.

The association between degree of nutrient intakes and SES was examined using multivariable regression for dichotomous outcome. Logistic regression models were used to examine the proportion of higher SES by quintiles of energy-adjusted nutrient intakes, after taking age, gender, race/ethnicity and household size into account. Proportions were expressed at mean age (56 years) and mean calorie intake (1800 Kcal/d) for the sample.

To examine the association between SES and diet cost, multivariable regressions for dichotomous outcome were used. Diet cost, energy-adjusted using the method of residuals and converted into quintiles, was used as the independent variable. Proportion of higher SES was used as the dependent variable. Following past studies, higher SES was defined in all three ways – combined higher income and higher education, only higher income, and only higher education. Covariates for each included age, gender, race/ethnicity, household size and total calorie intake. Proportions of higher SES were expressed at mean age (56 years) and mean calorie intake (1800 kcal/d) for the sample.

All analyses were conducted using STATA 10.0 and p-value of 0.05 was used to indicate statistical significance.

## Results

### Participant characteristics

The characteristics of the sample are presented in **Table 1**. The majority of S.O.S participants were women (64%). Mean age was 56±14 y. The sample was predominantly White (85%), with 4% African Americans and 7% Asians. More than half of the participants (62%) had an annual household income ≥$50,000, and 57% were college graduates. Based on combined SES index, respondents with higher SES (using both income ≥50,000 and at least college graduates) were 43%. Mean calorie intakes from all foods and beverages were 1700±666 Kcal/d for women and 1982±771 Kcal/d for men.

**Table 1:** Characteristics of the study sample.

Characteristics	Total
Gender	
Men	470 (36%)
Women	825 (64%)
Race/ethnicity	
Non Hispanic Whites	1088 (85%)
Non Hispanic Blacks	57 (4%)
Asians	90 (7%)
Others	47 (4%)
Income	
<50,000	433 (38%)
50,000−<100,000	408 (36%)
≥100,000	301 (26%)
Education	
High school or less	221 (17%)
Some college	330 (26%)
College graduates or higher	738 (57%)
SES combined index	
Income <50,000 and <college graduates	258 (23%)
Income <50,000 and ≥college graduates	173 (15%)
Income ≥50,000 and <college graduates	218 (19%)
Income ≥50,000 and ≥college graduates	490 (43%)

sum may not add up to 100% due to missing values.

### Quintiles of energy-adjusted nutrient intakes and diet cost

**Table 2** shows the relation between energy-adjusted nutrient intakes and diet cost. For vitamins A, C, D, E, B12, beta carotene, folate iron, calcium, potassium, magnesium, and fiber, lower quintiles of nutrient intakes were associated with significantly lower diet costs. The differences between the lowest and the highest quintile ranged from 9% to 40% after adjusting for calories and other covariates. The most pronounced differences were seen for vitamin C, beta carotene, potassium and magnesium. The lowest cost gradient was observed for vitamins D and E, folate, iron and calcium. The trends were all significant, with *P* for linear trend <0.0001 for each nutrient.

**Table 2:** Mean diet cost by quintiles of energy-adjusted nutrient intakes, adjusting for other covariates.^1^

Independent variables^2^	Mean diet cost by quintiles of energy-adjusted nutrient intakes										% change from Q1 to Q5	*P* 3
	Q1		Q2		Q3		Q4		Q5			
	Mean diet cost (95% CI)		Mean diet cost (95% CI)		Mean diet cost (95% CI)		Mean diet cost (95% CI)		Mean diet cost (95% CI)			
Vitamin A	8.3	(7.8, 8.7)	8.6	(8.2, 9.0)	9.1	(8.7, 9.5)	9.6	(9.2, 10.0)	10.2	(9.7, 10.6)	22.9	<0.0001
Vitamin C	8.0	(7.6, 8.4)	8.7	(8.3, 9.1)	9.3	(8.9, 9.7)	9.8	(9.4, 10.2)	10.5	(10.1, 10.9)	31.3	<0.0001
Vitamin D	8.6	(8.2, 9.0)	9.2	(8.7, 9.6)	9.3	(8.9, 9.7)	9.6	(9.2, 10.0)	9.8	(9.3, 10.4)	14.0	<0.0001
Vitamin E	8.7	(8.2, 9.2)	9.1	(8.6, 9.4)	9.4	(8.9. 9.8)	9.5	(9.1, 9.9)	9.7	(9.3, 10.2)	11.5	<0.0001
Vitamin B12	8.5	(8.1, 9.0)	9.1	(8.7, 9.5)	9.3	(8.9, 9.7)	9.7	(9.3, 10.1)	10.1	(9.6, 10.5)	18.8	<0.0001
Beta carotene	8.0	(7.5, 8.4)	8.7	(8.3, 9.1)	9.0	(8.6, 9.4)	9.6	(9.2, 10.0)	10.4	(10.0, 10.8)	30.0	<0.0001
Folate	8.6	(8.2, 9.0)	9.3	(8.8, 9.8)	9.4	(9.0, 9.8)	9.5	(9.1, 10.0)	9.6	(9.2, 10.1)	11.6	<0.0001
Iron	8.7	(8.2, 9.2)	9.1	(8.6, 9.5)	9.5	(9.0, 9.9)	9.6	(9.2, 10.0)	9.5	(9.1, 10.0)	9.1	<0.0001
Calcium	8.7	(8.2, 9.2)	8.9	(8.5, 9.3)	9.2	(8.8, 9.6)	9.4	(9.0, 9.8)	9.5	(9.0, 10.0)	9.2	<0.0001
Potassium	7.6	(7.2, 7.9)	8.3	(7.9, 8.6)	8.8	(8.5, 9.2)	9.7	(9.3, 10.0)	10.6	(10.2, 11.0)	39.5	<0.0001
Magnesium	7.8	(7.4, 8.2)	8.6	(8.2, 9.0)	9.1	(8.7, 9.5)	9.4	(9.0, 9.8)	10.6	(10.1, 11.0)	35.9	<0.0001
Fiber	8.3	(7.8, 8.7)	8.6	(8.2, 9.1)	8.9	(8.5, 9.3)	9.5	(9.1, 9.9)	10.2	(9.8, 10.6)	22.9	<0.0001
Saturated fats	10.6	(10.2, 11.1)	9.9	(9.5, 10.3)	9.5	(9.0, 9.9)	8.7	(8.3, 9.1)	8.1	(7.7, 8.5)	−23.6	<0.0001
Trans fats	10.2	(9.7, 10.6)	9.7	(9.2, 10.1)	9.1	(8.7, 9.5)	8.5	(8.1, 8.9)	8.1	(7.7, 8.6)	−20.6	<0.0001
Added sugars	9.7	(9.2, 10.1)	9.5	(9.1, 9.9)	9.4	(9.0, 9.9)	9.1	(8.7, 9.6)	8.5	(8.1, 9.0)	−12.3	<0.0001

Abbreviations: Q1, Quintile 1; Q2, Quintile 2; Q3, Quintile 3, Q4, Quintile 4; Q5, Quintile 5; CI, Confidence interval.

1 Adjusted for age, gender, race/ethnicity and total calorie intake. Presented at mean age of 56 years and mean calorie intake of 1800 Kcal/d for the sample.

2 Used as independent variables. Each nutrient (with the exception of fats and added sugar) was energy-adjusted using residual method and then converted into quintiles. For saturated fats, trans fats and added sugars, expressed as percent of total calories and then converted into quintiles.

3 Two sided *P* for trend test across quintiles of each independent variable.

On the other hand, persons in highest quintile of intakes for saturated fats, trans fats and added sugar had significantly lower diet costs, as compared to those in the lowest intake quintiles. The difference across extreme quintiles was as high as 23%. The trends were all significant, with *P* for linear trend <0.0001.

**Figure 1:**
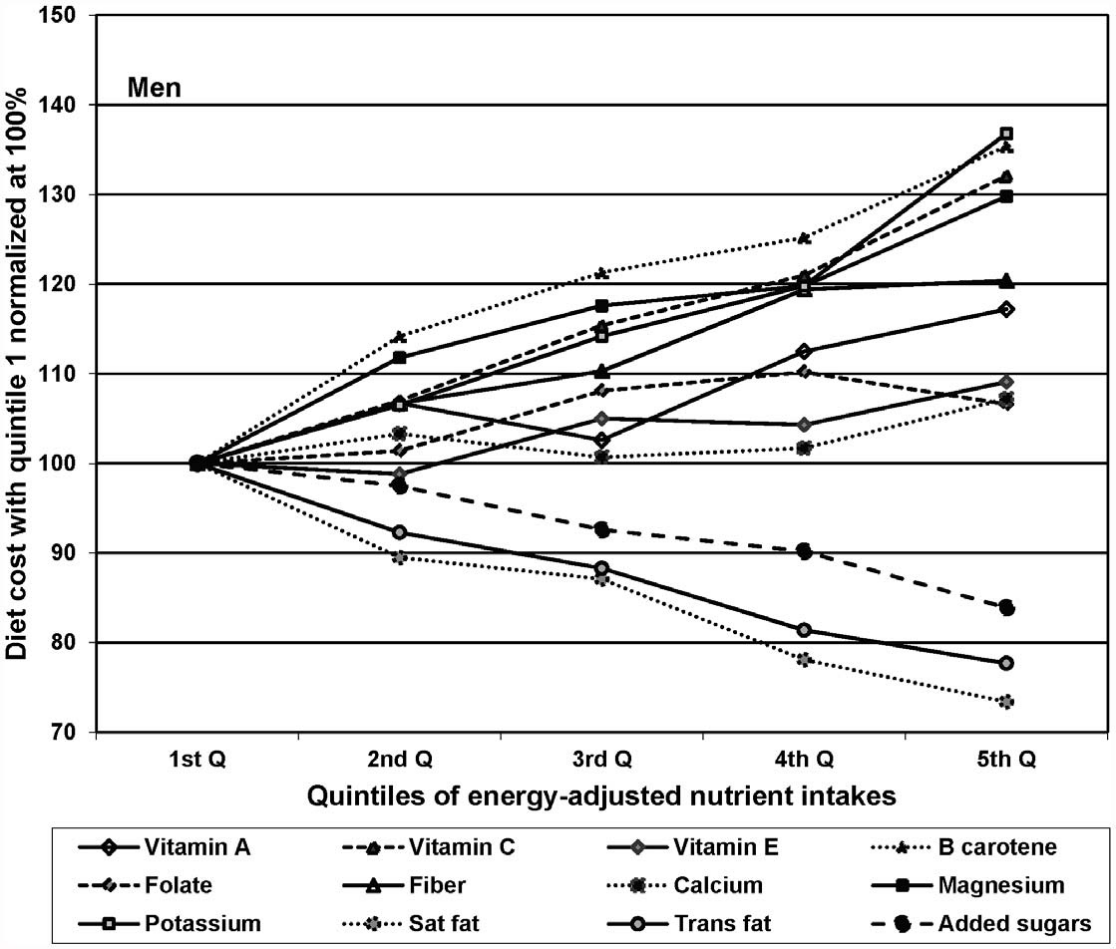
Diet cost by quintiles of energy-adjusted nutrient intakes, among men: results from multivariable regression.

Separate analyses of nutrient intakes with diet cost by gender are presented in **Figure 1** **and** **2**. The trends for men and women were all in the same direction; however the effects for most of the nutrients were stronger for women than for men.

**Figure 2:**
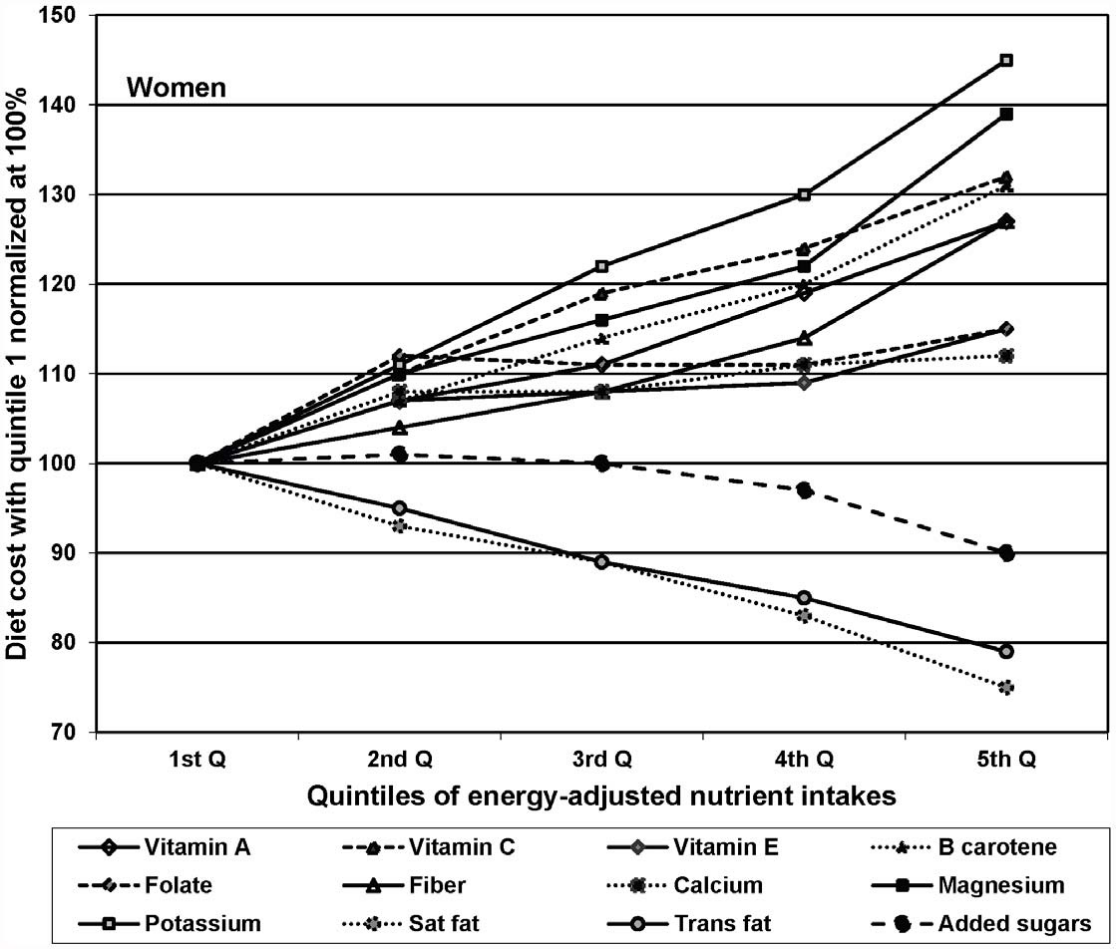
Diet cost by quintiles of energy-adjusted nutrient intakes, among women: results from multivariable regression.

### Quintiles of energy-adjusted nutrient intakes and socioeconomic factors

**Table 3** shows that energy-adjusted nutrient intakes also followed a strong SES gradient. Persons consuming diets in the lower quintiles of vitamin C, E, beta carotene, potassium, magnesium and fiber were significantly more likely to be from lower income and education groups, as compared to persons in the higher quintile of nutrient intakes. By contrast, those in higher quintiles of saturated and trans fats were associated with significantly lower SES. The trends were all significant with p-value <0.0001.

**Table 3:** Proportion of higher SES^1^ by quintiles of energy-adjusted nutrient intakes^2^, adjusting for other covariates.^3^

Independent variables	Proportion of higher SES by quintiles of energy-adjusted nutrient intakes										% diff from Q1 to Q5	*P* 4
	Q1		Q2		Q3		Q4		Q5			
	% higher SES (95% CI)		% higher SES (95% CI)		% higher SES (95% CI)		% higher SES (95% CI)		% higher SES (95% CI)			
Vitamin A	39.0	(26.5, 53.3)	38.5	(25.9, 52.9)	38.5	(26.5, 52.2)	41.2	(28.3, 55.3)	44.2	(30.9, 58.4)	13.3	0.236
Vitamin C	26.5	(16.6, 39.5)	36.7	(24.4, 51.1)	38.4	(25.7, 52.9)	53.3	(38.9, 67.1)	47.1	(32.9, 61.7)	77.7	<0.0001
Vitamin D	39.3	(27.3, 53.9)	41.1	(28.2, 55.3)	37.4	(25.5, 51.2)	40.5	(27.9, 54.4)	44.7	(31.2, 59.1)	13.7	0.398
Vitamin E	33.7	(22.4, 47.4)	35.0	(23.3, 49.2)	42.2	(28.9, 56.6)	42.2	(29.5, 56.0)	47.7	(34.1, 61.5)	41.5	<0.0001
Vitamin B12	43.9	(31.0, 57.7)	33.4	(22.0, 47.1)	35.8	(24.0, 49.8)	43.5	(30.4, 57.7)	46.6	(32.9, 60.8)	6.1	0.134
Beta Carotene	28.1	(17.7, 41.5)	37.6	(25.5, 51.5)	50.6	(36.3, 64.8)	42.1	(29.2, 56.0)	47.7	(34.0, 61.8)	69.8	<0.0001
Folate	36.3	(24.4, 49.9)	39.9	(27.2, 54.1)	41.9	(29.0, 56.1)	38.9	(26.1, 53.5)	46.9	(33.4, 60.8)	29.2	0.060
Iron	38.8	(26.5, 52.7)	41.0	(28.1, 55.2)	43.7	(30.5, 57.8)	40.2	(27.6, 54.2)	38.9	(26.5, 53.1)	0.2	0.954
Calcium	33.4	(21.5, 47.8)	37.2	(25.1, 51.2)	41.7	(28.9, 55.7)	41.0	(28.2, 55.1)	43.1	(30.0, 57.2)	29.0	0.032
Potassium	29.0	(18.2, 42.7)	32.4	(21.1, 46.3)	41.8	(28.6, 56.3)	46.1	(32.2, 60.6)	47.6	(33.7, 61.8)	64.1	<0.0001
Magnesium	29.2	(18.5, 43.2)	36.7	(24.6, 50.8)	36.8	(24.7, 50.9)	40.5	(27.7, 54.8)	53.8	(39.6, 67.5)	84.2	<0.0001
Fiber	29.0	(18.3, 42.6)	37.6	(25.3, 51.9)	40.0	(27.3, 54.3)	45.1	(31.6, 59.4)	46.3	(32.8, 60.2)	59.7	<0.0001
Saturated fats	51.8	(37.8, 65.7)	41.9	(28.7, 56.3)	41.9	(28.7, 56.4)	41.4	(28.2, 55.9)	29.2	(19.0, 4.6)	−43.6	<0.0001
Trans fats	54.7	(40.6, 68.1)	40.2	(27.3, 54.5)	38.4	(25.9, 52.8)	34.7	(23.1, 48.5)	21.0	(12.7, 32.4)	−61.6	<0.0001
Added Sugars	45.8	(32.4, 59.8)	37.8	(25.5, 51.8)	43.4	(30.3, 57.4)	39.5	(26.9, 53.6)	34.9	(23.3, 48.7)	−23.7	0.061

1 Higher SES used as the dependent variable. Indicate those with income ≥50,000 and at least college graduates.

2 Used as independent variables. Each nutrient (with the exception of fats and added sugar) was energy-adjusted using residual method and then converted into quintiles. For saturated fats, trans fats and added sugars, expressed as percent of total calories and then converted into quintiles.

3 Adjusted for age, gender, race/ethnicity, household size and total calorie intake. Proportions presented for mean age of 56 years and calorie intake of 1800 kcal/d.

4 Two sided *P* for trend test across energy-adjusted quintiles of each nutrient intake.

### Quintiles of energy-adjusted diet cost and socioeconomic factors

**Table 4** shows the association between energy-adjusted diet cost and measures of SES, after adjusting for covariates. Persons in lower quintiles of diet cost were significantly less likely to be from higher SES. The trends remained the same for all SES indicators: income, education or combined. The trends were all significant with *P*<0.0001.

**Table 4:** Proportion of higher SES by quintiles of energy-adjusted diet cost, adjusting for covariates.^1^

Dependent variables	Quintiles of energy-adjusted diet cost										% diff from Q1 to Q5	*P* 2
	Q1		Q2		Q3		Q4		Q5			
**Socioeconomic Indicators** 3												
% higher SES (income ≥50,000 and college degree or higher) (95% CI)	25.3	(15.7, 38.3)	31.7	(20.2, 46.1)	37.5	(25.1, 51.8)	44.8	(31.2, 59.1)	57.4	(42.9, 70.7)	126.8	<0.0001
% with higher income (income ≥ 50,000) (95% CI)	33.5	(21.2, 48.7)	38.1	(24.9, 53.4)	50.2	(35.0, 65.4)	60.2	(44.6, 73.8)	65.0	(50.2, 77.4)	94.0	<0.0001
% of higher education (college degree or higher) (95% CI)	48.7	(35.8, 61.5)	59.3	(46.2, 71.7)	64.3	(51.5, 75.2)	69.9	(57.9, 79.7)	78.4	(68.0, 86.1)	60.9	<0.0001

Abbreviations: Q1, Quintile 1; Q2, Quintile 2; Q3, Quintile 3, Q4, Quintile 4; Q5, Quintile 5; CI, Confidence interval; *P*, p-value; β, Beta coefficient; SD, Standard Deviation.

1 Adjusted for age, gender, race/ethnicity, household size and total calorie intake. Standardized at mean age of 56 years and mean calorie intake of 1800 kcal/d for the sample.

2 Two sided p-value for trend test across energy-adjusted quintiles of daily diet cost.

3 Higher SES (either income <5,000 K, or less than college education, or both as the reference category), Higher income (<50,000 as the reference category), Higher education (≤some college as the reference category).

## Discussion

Observational studies have established consistent associations between degrees of nutrient intakes and health outcomes. The present study, for the first time, examined degrees of nutrient intakes, for every key nutrient in the diet, in relation to estimated diet cost and participant SES. The study took care to follow standard epidemiological adjustment and stratification techniques.

There were significant findings. First, lower intakes of beneficial nutrients were associated with lower diet costs. Study respondents with lowest intakes of dietary fiber, vitamins A, C, D, E, and B12, beta carotene, folate, iron, calcium, potassium, and magnesium were also those who had lowest estimated diet costs. Coincidentally, some of these nutrients have been identified as nutrients of concern by the 2010 Dietary Guidelines [26], [34]. By contrast, higher intakes of fats and added sugars, typically associated with adverse health outcomes, were associated with lower diet costs. These are the nutrients to limit, as identified by the 2010 dietary guidelines [26]. Based on current eating habits, compliance with dietary guidelines is likely to entail higher diet costs for the consumer.

Second, persons with lower cost lower quality diets were more likely to be from lower SES groups. These findings are consistent with the existing literature on SES and diet quality [56] and diet cost [40], [44].

However, not all beneficial nutrients were equally expensive. The most pronounced positive gradient with diet cost was seen for vitamin C, beta carotene, potassium and magnesium – nutrients primarily obtained from fruits and vegetables. By contrast, calcium and vitamin D showed a weaker associations with diet cost, likely because milk and milk products are relatively inexpensive [57], [58]. Iron and folate also showed a weak association with diet cost, which may reflect the ubiquity and relatively low cost of grain products fortified with iron and folate. Further, gender differences were observed in some of these associations. Women with higher intakes of certain beneficial nutrients such as potassium and magnesium tend to have significantly higher diet costs as compared to men. This could be attributed to overall higher intakes of such nutrients per kcal among women than men, and that women also tend to choose more expensive sources of such nutrients. A recent study based on national level health survey found that women tend to have higher consumption of fruits and vegetables while men consume more meats [59]. Consistent findings were obtained in the present sample (results not shown).

There is clearly a need to identify and promote inexpensive food sources of key nutrients in order to improve the dietary quality of lower SES groups. A recent analysis of the 4 shortfall nutrients in the US diets showed that, in the context of current eating habits, complying with potassium guidelines was a particular challenge [60].

The present study had certain limitations. First, estimates of nutrient intakes and diet cost were each based on FFQs, which has certain known biases [42], [61], [62]. However, it is a useful tool to make comparisons across subjects and has been widely used in nutritional epidemiological studies. Second, diet cost estimates do not represent actual expenditures made by the study sample. Instead, these represent the lowest monetary value of the diet at which foods were available in the key retail supermarkets in the Puget Sound area. This method of estimating diet cost, in fact, offered certain advantages: a) it did not allow variation in diet cost, among individuals, simply due to differences in price of the same food item across stores, or due to differences in the amount spent while eating out, b) the use of retail food prices to calculate individual diet cost is the only method of estimating diet cost in the existing literature [40], [50], [63]–[66] and opens the door to individual level studies on diet cost, diet quality and health. Third, the average calorie intakes observed in the present sample were lower. However, this could be attributed to higher proportion of older adults (mean age of the present sample was 56 years) as these values were comparable to calorie intake estimates observed in National Health and Nutrition Examination Surveys (NHANES, 2001–08) and other health studies for that age, particularly for women. Fourth, the present study was based on cross sectional data, hence, associations observed between SES, diet cost and nutrient intakes cannot be causally interpreted.

Nonetheless, the present findings have implications for future research. First, diet cost variable ought to be taken into account in future studies on diets and disease risk. Second, further research is needed to identify cheaper ways of promoting beneficial nutrients to the consumer, particularly among lower income and lower education group.
